# A New Method for Modeling the Cyclic Structure of the Surface Microrelief of Titanium Alloy Ti6Al4V After Processing with Femtosecond Pulses

**DOI:** 10.3390/ma13214983

**Published:** 2020-11-05

**Authors:** Volodymyr Hutsaylyuk, Iaroslav Lytvynenko, Pavlo Maruschak, Volodymyr Dzyura, Georg Schnell, Hermann Seitz

**Affiliations:** 1 Institute of Robots and Machine Design, Military University of Technology, Gen. S. Kaliskiego str. 2, 00-908 Warsaw, Poland; 2 Department of Industrial Automation, Ternopil National Ivan Puuj Technical University, Ruska str. 56, 46001 Ternopil, Ukraine; d_e_l@i.ua (I.L.); maruschak.tu.edu@gmail.com (P.M.); volodymyrdzyura@gmail.com (V.D.); 3 Microfluidics, Faculty of Mechanical Engineering and Marine Technology, University of Rostock, Justus-von-Liebig-Weg 6, 18059 Rostock, Germany; georg.schnell@uni-rostock.de (G.S.); hermann.seitz@uni-rostock.de (H.S.)

**Keywords:** titanium alloy Ti6Al4V, implant microrelief, mathematical model, profilometry signals

## Abstract

A method of computer modeling of a surface relief is proposed, and its efficiency and high accuracy are proven. The method is based on the mathematical model of surface microrelief, using titanium alloy Ti6Al4V subjected to processing with femtosecond pulses as an example. When modeling the examples of microrelief, changes in the shape of segments-cycles of the studied surface processes, which correspond to separate morphological formations, were taken into account. The proposed algorithms were realized in the form of a computer simulation program, which provides for a more accurate description of the geometry of the microrelief segments. It was proven that the new method significantly increases the efficiency of the analysis procedure and processing of signals that characterize self-organized relief formations.

## 1. Introduction

It is known that during laser treatment of metals used for biomedical purposes (manufacture of implants), complex physicochemical processes take place in the surface layer, the kinetics of which is determined by the amount of energy introduced into the material and the processing time [1]. Periodic titanium patterns induce more uniform and direct cell growth. This effect is mainly connected with the surface properties of textured titanium implants [2,3]. When selected correctly, these parameters make it possible to form a surface layer of metals with a predetermined structure, grain size, phase composition, hardness and surface roughness [4]. However, estimating the parameters of a processed relief based on roughness alone is an approximate method that allows detecting the integral value of roughness only [5,6,7].

In this case, the geometric features of the relief formed, even with the same roughness values, may differ due to local peculiarities of micro- and macro-stresses distributed in the area of laser processing of materials [8,9]. Currently, investigations are ongoing into femtosecond laser treatment of titanium alloys, which is superior to the previously studied nanosecond laser treatment, as it makes it possible to create a surface topography of different geometries. It is known that increased overlap of laser-treated sections leads to an increased periodicity (cyclicity) of the treated surface microrelief. Femtosecond treatment of the material provides for the formation of highly ordered conical microstructures on the surface [10].

Modern methods of profilometry allow conducting functional diagnostics of the surface condition and detecting ordered structural formations. However, it remains important to create effective computer diagnostic systems for the automated processing of the received signals, followed by a preliminary diagnostic conclusion about the surface condition. Thus, the development of approaches for modeling the surface microgeometry of titanium alloys is the basis for creating a technology for making a self-organized microrelief with an optimal rate of osteointegration and modifying its surface with high-energy pulses [11,12,13].

Improving the methods of modeling the microgeometry of a laser-treated surface is one of the steps for ensuring the reproducibility of the relief formed and creating methods for describing its diagnostic state. Both parametric (roughness criteria) and nonparametric evaluation criteria are used to evaluate microgeometry. In particular, some works are known, the authors of which use densities and density functions describing the distribution of ordinates and tangents of the profile inclination angles, as well as profile microtopography. The effectiveness of the nonparametric approach to solving the optimization problems has been proven by numerous studies. In particular, earlier in [4], a mathematical model of the cyclic structure of the surface microrelief of the titanium alloy Ti6Al4V was described. The self-organization of the surface subjected to the impact-oscillatory laser effect was considered to be a cyclic random process, which provides for a description of the geometric features of the microrelief [14,15]. The components of the proposed model take into account the segments-cycles of cyclic microrelief.

The purpose of this research is to develop a new mathematical model that allows taking into account the amplitude parameters at each segment-cycle within the microrelief structure of the surface of titanium alloy Ti6Al4V and compare the results of the microrelief modeling using the new and known mathematical models.

## 2. Materials and Methods

**Figure 1:**
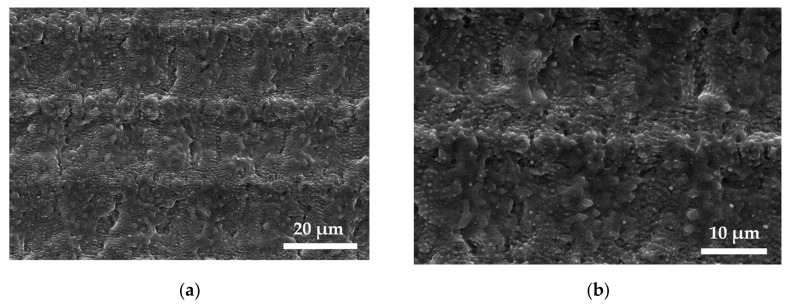
SEM images of structured Ti6Al4V surface with a laser pulse overlap of 50% and scanning line overlap of 40% at a fluence of q = 4.91 J/cm^2^. (**a**) A clear formation of trenches and ridges can be observed as a result of a low scanning line overlap (**b**) [4,10].

A model for creating relief formations on the surface of titanium alloy Ti6Al4V, which were polished to a roughness s = 0.065 ± 0.003 μm and treated with a laser, was considered in this article [4,10]. A Yb-doped fiber laser system of the type UFFL_60_200_1030_SHG from Active Fiber Systems GmbH, Jena, Germany, with a pulse duration of 300 fs was used in this research. The system enabled a pulse repetition rate from 50.3 kHz up to 18.6 MHz with a maximum pulse energy of 200 µJ. The linear polarized Gaussian beam was deflected by an intelliSCAN 14 scan head (Scanlab GmbH, Puchheim, Germany) and focused with an f-theta lens with a focal length of 163 mm, resulting in a circular focus diameter of *d_f_* = 36 µm (1/e^2^). For the experimental work, a wavelength of 1030 nm was applied. Laser structuring was carried out on a Microgantry GU4 micromachining center from Kugler GmbH, Salem, Germany. This spot diameter was used for all laser parameter calculations [4,10]. The findings presented in article [4], in which pulse treatment was performed by overlapping the areas of laser treatment (LO), were the experimental basis of this research. High values of the parameters considered caused phase transformations and heat accumulation, which led to a reduced ablation threshold and increased roughness. In this paper, the surface relief properties of titanium alloy Ti6Al4V were modeled by LO 40% at a laser fluence of *q* = 4.91 J/cm^2^, Figure 1, to verify the method efficiency. Both trenches and ridges were strongly covered with melt and nanoprotrusions due to a high level of fluence far above the ablation threshold of the material, resulting in a pronounced heat accumulation [4,10].

A generalized theoretical and methodological approach, which consisted of identifying the segmental structure of cyclic signals with a variable rhythm, was applied to the analysis of the surface relief, allowing us to process experimental data as part of the stochastic approach.

## 3. A New Mathematical Model of Cyclic Microrelief

The profilogram of the surface microrelief of the titanium alloy Ti6Al4V after femtosecond pulse treatment was considered as a stochastic cyclic process. In [14], the definition of a cyclic random process is given. It is a separable random process $\xi \left(\omega ,l\right),\omega \in \Omega ,l\in \left[0,L\right)$, which is called a cyclic random process of a continuous argument, provided that there is the function $T\left(l,n\right)$, which satisfies the conditions of the rhythm function. In addition, finite-dimensional vectors ($\xi (\omega ,l_{1})$, $\xi (\omega ,l_{2})$,…, $\xi (\omega ,l_{k})$) and ($\xi (\omega ,l_{1}+T\left(l_{1},n\right))$, $\xi (\omega ,l_{2}+T\left(l_{2},n\right)))$,..., $\xi (\omega ,l_{k}+T\left(l_{k},n\right)$), n∈Z, where $\left\{l_{1},l_{2},\ldots ,l_{k}\right\}$ is the set of the process separability, $\xi \left(\omega ,l\right),\omega \in \Omega ,l\in \left[0,L\right)$ are stochastically equivalent in a broad sense for all integers k∈N where $T\left(l,n\right)$ is the rhythm function of the cyclic process, which reflects the regularities in the variation of temporal (spatial, in our case) intervals between its single-phase values. The main properties of this function are described in [15].

**Figure 2:**
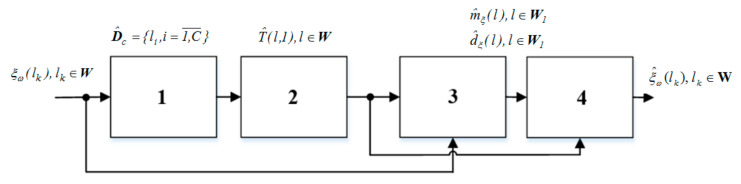
Block diagram of the computer modeling of the microrelief process (known approach): 1—assessment of the segmental structure of the microrelief; 2—assessment of the rhythmic structure of microrelief; 3—statistical processing of microrelief; 4—cyclic microrelief modeling.

Figure 2 presents a block diagram of the well-known approach to modeling the surface microrelief [14]. Accordingly, the mathematical model of the cyclic surface microrelief was presented in a form that takes into account its segmental cyclic structure:

(1) $$\xi_{\omega }(l)=\sum_{i=1}^{C}f_{i}\left(l\right),\;l\in W$$

where C is the number of segments-cycles of the cyclic microrelief, W is the definition area of the cyclic microrelief, while the region of its values in case of the stochastic approach is the Hilbert space of random variables given on a single probabilistic space $\left(\xi_{\omega }\left(l\right)\in \Psi =L_{2}(\Omega ,P)\right)$. In expression (1), segments-cycles of the cyclic microrelief process are determined by indicator functions, that is:

(2) $$f_{i}\left(l\right)=\xi_{\omega }\left(l\right)\times I_{W_{i}}\left(l\right),\;i=\bar{1,C},\;l\in W$$

In this case, the indicator functions, which allocate segments-cycles, were defined as:

(3) $$I_{W_{i}}\left(l\right)=\left\{\begin{array}{c} 1,\;l\in W_{i},\\ 0,\;l\notin W_{i}. \end{array}\right.,\;i=\bar{1,C}$$

where $W_{i}$ is the definition area of the indicator function, which in case of a discrete signal is W=D, that is, equals a discrete set of samples.

(4) $$W_{i}=\left\{l_{i,j},j=\bar{1,J}\right\},\;i=\bar{1,C},$$

The segmental cyclic structure ${\hat{D}}_{c}$ is taken into account by a set of spatial samples $\left\{l_{i}\right\}$ or $\left\{l_{i,j}\right\}$, $i=\bar{1,C}$, $j=\bar{1,J}$. The mathematical model (1) takes into account the rhythm of the cyclic microrelief using the continuous rhythm function $T\left(l,n\right)$, namely:

(5) $$I_{W_{i}}\left(l\right)=I_{W_{i+n}}\left(l+T(l,n)\right),\;i=\bar{1,C},\;n=1,\;l\in W.$$

In order to assess the rhythm function $T\left(l,n\right)$, the segmental structure of the microrelief (in this case, the segmental cyclic structure) was determined as ${\hat{D}}_{c}=\{l_{i},\;i=\bar{1,C}\}$, which is a set of spatial moments that correspond to the boundaries of the segments-cycles of the microrelief.

Having obtained the segmental structure ${\hat{D}}_{c}$ and estimated the rhythmic structure (discrete rhythm function $T\left(l,n\right)$), we used the methods of statistical processing. As a result, we obtained statistical estimates of probabilistic characteristics (mathematical expectation ${\hat{m}}_{\xi }(l),\;l\in W_{1}$ and dispersion ${\hat{d}}_{\xi }(l),\;l\in W_{1}$) of the cyclic microrelief process. After this, the obtained information was used for computer simulation of the realization of the cyclic process of surface microrelief ${\hat{\xi }}_{\omega }(l_{k}),\;l_{k}\in W$.

**Figure 3:**
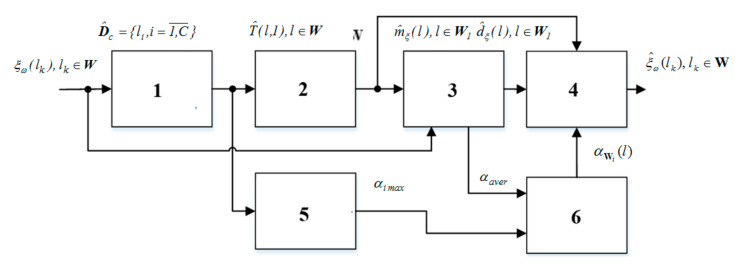
Block diagram of the computer modeling of the microrelief process (new approach): 1—assessment of the segmental structure of the microrelief; 2—assessment of the rhythmic structure of microrelief; 3—statistical processing of microrelief; 4—cyclic microrelief modeling; 5—determination of maximums of segments-cycles of microrelief; 6—estimation of the scale factors of the microrelief amplitude.

For an adequate description of the real process, the microrelief amplitudes on the segments-cycles need to be considered (this was prevented by the mathematical model presented in [14]); therefore, we take them into account in the new mathematical model, Figure 3.

In the new model (1), segments-cycles of the cyclic microrelief process are defined as multiplicative components, taking into account the indicator functions and scale coefficients of the microrelief amplitude, that is:

(6) $$f_{i}\left(l\right)=\xi_{\omega }\left(l\right)\cdot \alpha_{W_{i}}(l)\cdot I_{W_{i}}\left(l\right),\;i=\bar{1,C},\;l\in W.$$

In Formula (6), an additional component $\alpha_{W_{i}}(l)$ is introduced, which reflects the scale factors of the microrelief amplitude on each segment-cycle of the cyclic process, namely:

(7) $$\alpha_{W_{i}}\left(l\right)=\left\{\begin{array}{c} \alpha_{i},\;l\in W_{i},\\ 0,\;l\notin W_{i}. \end{array}\right.,\;i=\bar{1,C},$$

where $\alpha_{i}$ are the scale coefficients of the microrelief amplitude on each i-th segments-cycles defined as follows:

(8) $$\alpha_{i}=\frac{\alpha_{i\;\max}}{\alpha_{aver}},\;i=\bar{1,C},$$

where $\alpha_{i\;\max}$ is the maximum value of the microrelief amplitude on the i-th segment-cycle (determined at the segmentation stage of the cyclic microrelief process), $\alpha_{aver}$ is the average value of the microrelief amplitude (the maximum value of the mathematical expectation amplitude determined at the stage of statistical processing of the cyclic microrelief process).

## 4. Modeling Results

**Figure 4:**
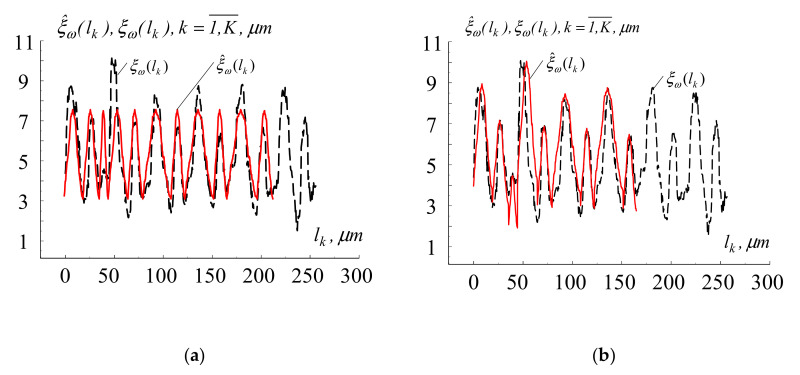
The result of modeling the surface microrelief of titanium alloy Ti6Al4V after processing with femtosecond pulses: (**a**) based on the well-known mathematical model [14]; (**b**) based on the proposed mathematical model (red line—experimental data; black line—results of modeling).

A comparative analysis of the results of computer modeling was performed. It showed that the new method of computer modeling of microrelief was more accurate than the well-known method presented in [14]. This is because the new method is based on a mathematical model of the self-ordered relief, which is presented as a cyclic random process, taking into account the amplitude parameters on each segment-cycle, Figure 4. Higher accuracy of computer modeling was achieved due to adapting the description of the shape of the segments-cycles under study.

This approach eliminated the negative effect of taking into account only the statistical evaluation (mathematical expectation) in case of modeling the relief morphology in the presence of a significant height variation of the microrelief elements.

**Figure 5:**
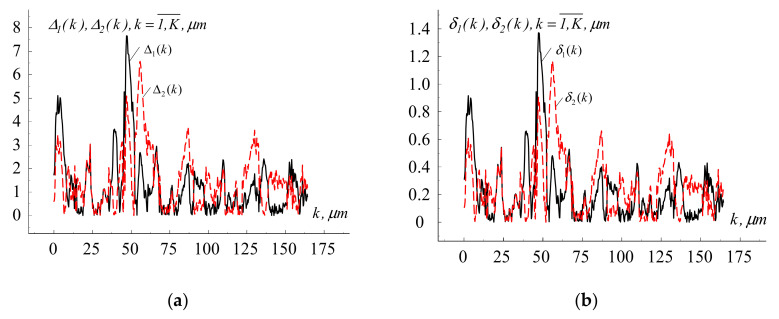
Fragments of the absolute and relative errors of computer modeling of the surface microrelief of titanium alloy Ti6Al4V after processing with femtosecond pulses: (**a**) absolute errors, (**b**) relative errors.

**Estimation of errors.** In order to compare the proposed and well-known mathematical models, computer modeling of microrelief realizations was performed, and modeling results were estimated by defining the absolute $\Delta_{q}(k)$ and relative errors $\delta_{q}(k)$, Figure 5.

(9) $$\Delta_{q}(k)=\sqrt{\frac{1}{K}\sum_{k=1}^{K}{\left(\xi_{\omega }(l_{k})-{\hat{\xi }}_{\omega }(l_{k})\right)}^{2}},\;q=\bar{1,2},\;k=\bar{1,K},\;l_{k}\in W,$$

(10) $$\delta_{q}(k)=\frac{\Delta_{q}(k)}{\sqrt{\frac{1}{K}\sum_{k=1}^{K}{\left({\hat{\xi }}_{\omega }(l_{k})\right)}^{2}}},\;q=\bar{1,2},\;k=\bar{1,K},\;l_{k}\in W.$$

where ${\hat{\xi }}_{\omega }(l_{k})$ is the computer-simulated realization of the cyclic microrelief process (one of the two approaches); $\xi_{\omega }(l_{k})$ is the experimentally obtained realization of the cyclic microrelief process; computer simulation errors based on the well-known model were identified at *q* = 1; computer simulation errors based on the new model were identified at *q* = 2 (new approach).

It was found that the relative root mean square modeling error for the studied case (new approach) did not exceed 1.2 (12%). It should be noted that the comparative analysis of the relief reproduction accuracy was individual for each realization of the relief formation process, Table 1.

Based on a new mathematical model of the surface relief, as well as previously developed approaches to estimating the segmental and rhythmic structures and probabilistic characteristics of the process [19], a new approach to computer modeling of the cyclic microrelief realization on implant surfaces has been substantiated [20,21].

## 5. Conclusions

Based on the new mathematical model of surface microrelief, as applied to titanium alloy Ti6Al4V after its processing with femtosecond pulses, a method of computer modeling of the surface microrelief realization was developed. The model contains the components that take into account changes in the shape of cycles-segments of the processes investigated. The developed method of computer modeling makes it possible to describe more precisely the features of microrelief segments. This allowed increasing the efficiency of their processing procedure and computer modeling in information systems.

The software for computer simulation of the surface microrelief realization created on the basis of the new model can be integrated into specialized software for technical diagnostics of the surface condition and modeling experiments conducted after the precision laser processing. Using the developed software, a series of experiments on the processing of real microrelief sections by the new and well-known methods was performed. The obtained results of comparative analysis of modeling errors using the new method confirmed its higher accuracy in describing the segments-cycles as compared to the well-known method.

In further scientific research, it is planned to modify the mathematical model so as to allow considering the features of the microrelief nonlinearity (presence of a trend) on curvilinear surfaces.

## Figures and Tables

**Table 1:** Comparison of characteristics of the well-known and new mathematical models of the surface relief after laser treatment.

Models	Taking into Account the Cyclical Nature of the Relief	Taking into Account the Random Nature of the Relief	Taking into Account the Morphological Features of Segments-Cycles of the Relief	Taking into Account the Rhythmic Features of the Deployment of Segments-Cycles of the Relief	Taking into Account the Amplitude Features of Segments-Cycles of the Relief
Well-known [5,6,7,16,17,18,19]	+	+	+/−	+	−
New	+	+	+	+	+

“+”—takes into account (reflects); “−”—does not take into account (does not reflect); “+/−”—partially takes into account (partially reflects).

## References

[1] Van Driel H.M., Sipe J.E., Young J.F. (1982). Laser-induced periodic surface structure on solids: A universal phenomenon. Phys. Rev. Lett..

[2] Vorobyev A., Guo C. (2007). Femtosecond laser structuring of titanium implants. Appl. Surf. Sci..

[3] Kuczyńska-Zemła D., Kijeńska-Gawrońska E., Pisarek M., Borowicz P., Swieszkowski W., Garbacz H. (2020). Effect of laser functionalization of titanium on bioactivity and biological response. Appl. Surf. Sci..

[4] Schnell G., Duenow U., Seitz H. (2020). Effect of laser pulse overlap and scanning line overlap on femtosecond laser-structured Ti6Al4V surfaces. Materials.

[5] Filimonova E.A. (2014). Development of the Methodology and Program of the Automated Control of Microgeometry of Surfaces of Details of Instruments Using Graphic Criteria and Their Use in Technological Research, C. Ph.D. Dissertation.

[6] Gibadullin I.N., Valetov V.A. (2019). Image of the surface profile as a graphic criterion of its roughness. J. Instrum. Eng..

[7] Zhou W., Tang J.Y., He Y.F., Zhu C.C. (2017). Modeling of rough surfaces with given roughness parameters. J. Cent. South Univ..

[8] Vorobyev A.Y., Guo C. (2012). Direct femtosecond laser surface nano/microstructuring and its applications. Laser Photon. Rev..

[9] Kuznetsov G.V., Feoktistov D.V., Orlova E.G., Batishcheva K., Ilenok S.S. (2019). Unification of the textures formed on aluminum after laser treatment. Appl. Surf. Sci..

[10] Schnell G., Polley C., Bartling S., Seitz H. (2020). Effect of chemical solvents on the wetting behavior over time of femtosecond laser structured Ti6Al4V surfaces. Nanomaterials.

[11] Varlamova O., Reif J., Varlamov S., Bestehorn M., Sakabe S., Lienau C., Grunwald R. (2015). Self-organized Surface Patterns Originating from Laser-Induced Instability. Progress in Nonlinear Nano-Optics. Nano-Optics and Nanophotonics.

[12] Romano J.-M., Garcia-Giron A., Penchev P., Dimov S. (2018). Triangular laser-induced submicron textures for functionalising stainless steel surfaces. Appl. Surf. Sci..

[13] Aguilar-Morales A.I., Alamri S., Kunze T., Lasagni A.F. (2018). Influence of processing parameters on surface texture homogeneity using direct laser interference patterning. Opt. Laser Technol..

[14] Lytvynenko I.V., Maruschak P.O. (2015). Analysis of the state of the modified nanotitanium surface with the use of the mathematical model of a cyclic random process. Optoelectron. Instrument. Proc..

[15] Lytvynenko I.V., Maruschak P.O., Lupenko S.A., Popovych P.V. (2016). Modeling of the ordered surface topography of statically deformed aluminum alloy. Mater. Sci..

[16] Frischer R., Krejcar O., Selamat A., Kuca K. (2020). 3D surface profile diagnosis using digital image processing for laboratory use. J. Cent. South Univ..

[17] Wang X., Feng C. (2002). Development of empirical models for surface roughness prediction in finish turning. Int. J. Adv. Manuf. Technol..

[18] Ozcelik B., Bayramoglu M. (2006). The statistical modeling of surface roughness in high-speed flat end milling. Int. J. Mach. Tools Manuf..

[19] Lytvynenko I.V., Maruschak P.O., Lupenko S.A., Hats Y.I., Menou A., Panin S.V. (2016). Software for segmentation, statistical analysis and modeling of surface ordered structures. AIP Conference Proceedings.

[20] Muller F., Kunz C., Graf S. (2016). Bio-inspired functional surfaces based on laser-induced periodic surface structures. Materials.

[21] Schnell G., Staehlke S., Duenow U., Nebe J.B., Seitz H. (2019). Femtosecond laser nano/micro textured Ti6Al4V surfaces—Effect on wetting and MG-63 cell adhesion. Materials.

